# Variation in Chemical, Textural and Sensorial Traits Among Remontant Red Raspberry (*Rubus idaeus* L.) Cultivars Maintained in a Double-Cropping System

**DOI:** 10.3390/plants13233382

**Published:** 2024-12-01

**Authors:** Slavica Spasojević, Vuk Maksimović, Dragica Milosavljević, Ilija Djekić, Dragan Radivojević, Ana Sredojević, Jasminka Milivojević

**Affiliations:** 1 Department of Fruit Science, Faculty of Agriculture, University of Belgrade, 11080 Belgrade, Serbia; slavica.spasojevic@agrif.bg.ac.rs (S.S.); draganr@agrif.bg.ac.rs (D.R.); 2 Department of Life Sciences, Institute for Multidisciplinary Research, University of Belgrade, 11030 Belgrade, Serbia; maxivuk@imsi.rs (V.M.); dragicar@imsi.rs (D.M.); 3 Department of Food Safety and Quality Management, Faculty of Agriculture, University of Belgrade, 11080 Belgrade, Serbia; idjekic@agrif.bg.ac.rs (I.D.); ana.sredojevic@agrif.bg.ac.rs (A.S.)

**Keywords:** primocane, floricane, sugars, organic acids, berry color, total quality index

## Abstract

Remontant raspberry cultivars originally produce fruit in the upper part of primocanes in the fall, but if retained over winter, they can produce a second crop in the lower part of the floricanes the following spring. Maintaining remontant cultivars to yield twice during the cane’s growth cycle corresponds to a double-cropping system, which enables an increase in the total yield and the extension of the fruiting season. To date, there is little information on changes in fruit quality between primocane and floricane crops. The aim of this study was, therefore, to investigate variations in the content of sugars and organic acids, fruit weight, color and textural and sensorial attributes among five newly introduced remontant raspberry cultivars (‘Dafne’, ‘Kokanee’, ‘Paris’, ‘Versailles’ and ‘Primalba’) and the control cultivar ‘Enrosadira’. The specific aim was to evaluate how a double-cropping system in each cultivar may affect the variability in quality traits between primocane and floricane crops. The results showed a significant increase in fruit weight and individual and total sugar content in primocane crops, while significantly brighter red-colored and firmer fruits were observed in floricane crops. Cultivars did not differ from the control regarding total sugar content and sweetness index, while the content of individual sugars caused greater variations. The highest content of citric, malic and total acid (9.74, 1.42 and 11.25 mg 100 g^−1^ FW, respectively) were recorded in ‘Paris’, by which this cultivar was the only one distinguished from the control. ‘Dafne’ and ‘Versailles’ exhibited better internal and external fruit quality on primocanes, having significantly larger fruits (6.83 g and 6.37 g, respectively) and twice the increased sugar content. The lowest fruit weight was observed in ‘Kokanee’ for both primocane (4.63 g) and floricane (3.65 g) crops. ‘Kokanee’ and ‘Primalba’ also performed worse than the control for most sensory attributes in both seasons. Based on the analysis of the overall fruit quality linked to the appearance-, texture- and taste-related attributes that affect consumer preference, cultivars ‘Enrosadira’, ‘Versailles’ and ‘Dafne’ stood out, while ‘Paris’ showed high uniformity in fruit quality between crops, but scored the worst according to the total quality index.

## 1. Introduction

Raspberry (*Rubus idaeus* L.) is the third most important berry crop, with a total world production of 947,852 t in 2022 [1]. The Republic of Serbia is among the world’s leading producers with a total cultivated area of 19,703 ha and an annual production of 116,093 t. In commercial raspberry plantations, the most common floricane-fruiting cultivars are ‘Willamette’ and ‘Meeker’, whose fruits are intended for freezing/processing (>90%) and export to the foreign market as various frozen products [2]. More recently, new remontant cultivars have been introduced into plantations to meet the growing demand of producers to extend the harvest period and allow off-season production of raspberries [3].

In contrast to floricane cultivars which produce fruit on biennial canes, remontant cultivars normally bear fruits in the upper part of annual canes (primocanes) during the fall of the first year and if these canes are retained for the following year, a second crop is produced from the buds in the lower part of biennial canes (floricanes) during the spring. In this manner, they can be maintained to produce two crops on the same canes, corresponding to a double-cropping cultivation system [4,5,6]. In addition to high yields of excellent quality fruit, the main advantage of remontant cultivars is the extension of the harvest season to guarantee a year-round supply of raspberries and cover the rising market demand [7]. Moreover, a double-cropping system enables an increase in cumulative yield and the possibility of programmed production under a protected environment. The development of new cultivation techniques, such as soilless culture, is also a new direction to produce year-round raspberries for the fresh market. This growing system allows controlled nutrient management, regulated plant growth and higher yield per unit area [8,9]. However, apart from the cultivation system, genotype remains the most important factor that affects the quality of the fruit [10,11]. Currently, ‘Enrosadira’ is the commonly grown primocane cultivar [8], which is highly productive for both primocanes and floricanes [12], making it suitable for growing in a double-cropping system.

Therefore, it can be challenging to introduce new high-quality raspberry cultivars with desirable quality parameters for the fresh market, such as large, firm fruit, high skin strength, glossy bright color, low acid content and high nutritional value [3], which altogether contributes to their attractive appearance and outstanding taste. The fruit taste is mostly determined by sugar and acid content and the ratio of these two, with the type and quantity of individual compounds reflecting changes in quality [13]. The main sugars in raspberries are fructose, glucose and sucrose, with twice as much fructose content [14], contributing to a greater sweetness index. The predominant organic acids are citric and malic, while fumaric and shikimic acids are less abundant [15]. Besides fruit taste, sugars and organic acids greatly influence changes during fruit ripening affecting consumer acceptability [16]. During the ripening process, biochemical, physical and sensory modification are reflected in changes in chemical composition, fruit softening and color development, whereby the degree of color development can indicate ripeness, and thus biochemical quality and sensory acceptability [17]. In addition to sweet and acidic taste as the most commonly used characteristics to describe the sensorial quality of raspberry fruit, other frequently used attributes are raspberry flavor, astringency, juiciness and seediness [18]. While taste traits are dependent on the sugar and organic acid content, the texture and color traits seem more uncertain because of the lack of precision in the instrumental measures and the high interactions among traits [19]. According to Villamor et al. [20], consumer preference was guided by flavor in combination with appearance, taste, texture and mouthfeel attributes. Regarding the effect of the cultivation system, better overall fruit quality of remontant raspberry cultivars has been previously found in a single fall crop management [21]. Conversely, Hanson et al. [4] reported consistent fruit quality during both cropping seasons within a genotype. In colder climates, due to a shorter season, which prevents fall harvest on primocanes, remontant raspberries are maintained only for summer cropping, showing excellent fruit quality in floricanes [6]. However, it is still unknown how new raspberry cultivars respond to a double-cropping system and whether there are differences in fruit quality between primocane and floricane crops [4]. Thus, the aims of this study were (i) to estimate variability among six remontant raspberry cultivars for the content of primary metabolites, color and textural and sensorial fruit traits and (ii) to evaluate how a double-cropping system in each cultivar may influence variability in quality traits between primocane crops in the fall and floricane crops in the spring of the following year.

## 2. Results and Discussion

### 2.1. Individual Sugars, Organic Acids and Sweetness Indices

The most abundant sugars found in samples of six remontant raspberry cultivars were glucose, fructose and sucrose, represented almost equally (Table 1), which is in line with previously published data by Stavang et al. [22].

In other studies, sucrose was not detected in raspberry samples [16], or lower concentrations were found in comparison to reducing sugars (glucose and fructose) [13,15], indicating an increased activity of invertase, the enzyme responsible for the hydrolysis of sucrose to glucose and fructose. Naturally, a strong correlation between the three sugars has been confirmed [15,23], while only sucrose is found to correlate with sweetness evaluated by consumers [22]. Still, higher fructose content is considered desirable, as it contributes to the sweetest taste. Similar to Forney et al. [14] and Titirica et al. [24], who found up to twice as much fructose in raspberry in comparison to glucose and sucrose content, in our study, fructose content was slightly elevated, representing 31–44% of the total of all detected sugars. Arabinose and myoinositol were also detected in trace amounts, accounting for 2–5% of sugars in raspberry fruit. Munoz-Almagro et al. [25] claimed to be the first to quantify myoinositol in raspberries in the amount of 1.1 mg g^−1^ of dry fruit weight. This result was previously found in green and unripe fruits, in the initial stages of development, but was not detected in the fully ripe stage [23]. This indicates that myoinositol content decreases during ripening, which can clarify the differences between fall and spring crops. In fact, polyol myoinositol oxidates in plant tissues producing glucuronic acid, a direct precursor of xylose, whose epimerization can further result in arabinose formation [26]. This explains an inverse dependency that can be observed between myoinositol and arabinose content. In our samples, arabinose content varied from 0.08 g to 0.49 g 100 g^−1^ FW, having generally higher values in comparison to the previous report in several floricane-fruiting cultivars [15].

**Figure 1:**
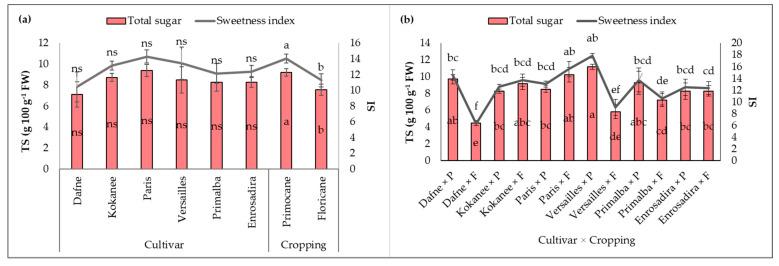
Total sugar content (TS) and sweetness index (SI) of six remontant raspberry cultivars: (**a**) effects of cultivar and cropping; (**b**) cultivar × cropping interaction. Data are presented as the means (*n* = 3) ± standard error and those designed by the same letter(s) (a–f) are not statistically different according to the LSD test at *p* ≤ 0.05. Differences caused by the cultivar are displayed in relation to the control cultivar ‘Enrosadira’; ns represent nonsignificant differences; FW—fresh weight.

Both factors, individually and in interaction, exposed statistically significant effects on individual sugar content and sweetness index (SI) (Figure 1) in raspberry samples. Despite significant differences regarding all individual sugars, the total sugar content (TS) (Figure 1) was not affected by the factor cultivar, while cropping and interaction caused significant variations at *p* ≤ 0.001. The greatest average content of TS (9.39 g 100 g^−1^ FW) and the highest SI (14.32) were detected in the cultivar ‘Paris’, while the lowest values were registered (7.1 g 100 g^−1^ FW and 10.45, respectively) in ‘Dafne’ indicating the least sweet taste. The cultivar ‘Dafne’ also deviated significantly from the control cultivar in the content of three main sugars (glucose, fructose and sucrose); however, all cultivars did not differ from ‘Enrosadira’ in terms of TS and SI.

The content of all individual sugars, except for arabinose, was significantly higher in primocane cropping, leading to a higher SI of the fruits harvested in the fall. However, cultivars ‘Enrosadira’, ‘Paris’ and ‘Kokanee’ showed the greatest uniformity in sugar content between the fall and spring crops. Conversely, ‘Dafne’ and ‘Versailles’ expressed significant variation in the content of all individual sugars, and up to 2-fold higher TS content was found in the fall crop (Figure 1b).

Besides sugars, taste attributes are also driven by organic acid content that gives the fruit its refreshingly fruity sour taste. In line with the previous findings [13,15], the most represented organic acids were citric and malic, reaching 87% and 12% of total acids (TAs) in raspberry fruit, respectively (Table 2). The content of the main organic acids varied significantly among the cultivars, with the greatest content of malic and citric acid and TA recorded in the cultivar ‘Paris’ (1.42, 9.74 and 11.25 mg g^−1^ FW, respectively); this cultivar differs from the control cultivar ‘Enrosadira’. Tartaric, shikimic and fumaric acids found in trace amounts were the only ones significantly affected by the cultivar × cropping interaction. On the contrary, variations were not found in the content of the most represented acids (citric and malic) and thus total organic acids. Shikimic acid was the only acid conditioned by cropping, with elevated concentrations in the fruit harvested on primocanes in all cultivars tested.

Quantified values of sugars and organic acids in our study are similar to reported values for the cultivar ‘Polka’, except for remarkably higher citric acid content [27], indicating a less balanced sugar/acid ratio. Higher concentrations of sugars and organic acids were also reported for different raspberry cultivars in other studies [15,22,28], but this may possibly be due to quantification in dried samples in contrast to our use of fresh fruit samples. Nevertheless, our fruit samples contained higher malic, fumaric and shikimic acids than detected in dried samples of the Bulgarian raspberry cultivar [23].

### 2.2. Fruit Color

**Figure 2:**
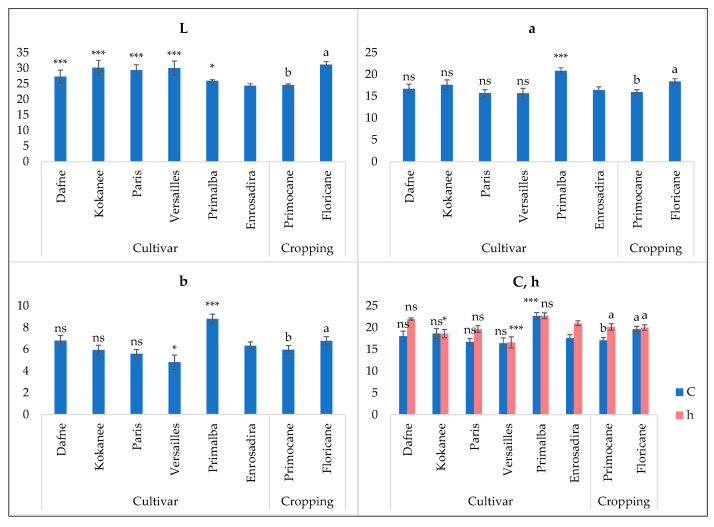
Effect of the cultivar and cropping season on CIE Lab color parameters of remontant raspberry cultivars in comparison to the control cultivar ‘Enrosadira’. L—lightness; a—redness; b—yellowness; h—hue angle or color shade; C—chroma or color saturation. Data are presented as the means (*n* = 3) ± standard error and those designed by the same letter(s) (a, b) are not statistically different according to the LSD test at *p* ≤ 0.05. Differences caused by the cultivar are displayed in relation to the control cultivar ‘Enrosadira’ and indicated as follows: ns, * and *** represent nonsignificant and significant differences at *p* ≤ 0.05 and *p* ≤ 0.001, respectively.

An important indicator of raspberry fruit quality is the color, which, along with sugars and organic acids, is an important attribute for consumer acceptance. Thus, sweetness and acidity were reported to depend on color at harvest, whereas lighter berries were perceived as having a more acidic taste [22]. In our samples, fruit lightness (L) was significantly affected by both factors and interaction at *p* ≤ 0.001 (Figure 2; Table 3). This caused the deviation of all cultivars from the control sample (Figure 2).

The highest L values were found in ‘Versailles’ (30.21) and ‘Kokanee’ (30.03), indicating the brightest color of the fruit, while ‘Enrosadira’ stood out as the darkest (24.45) (Figure 2). However, our fruit samples were generally brighter than reported previously [10,29], which is considered preferable for the fresh market. All other parameters, except for hue angle, were significantly affected by cultivar and cropping season. In our study, h ranged from 15.09 to 23.13 (Table 3), which was slightly lower than determined by Mazur et al. [10] and Aaby et al. [29], corresponding to a more red-bluish color. The fruit of the ‘Primalba’ cultivar, on average, exhibited the reddest (a), yellowest (b), most saturated (C) and intense shade color (h). Higher L*a*b* and chroma values were detected in spring cropping on floricanes, indicating a brighter, more red and saturated color. Additionally, a significant interaction effect was found for L and a, whereas all cultivars except for ‘Primalba’ and ‘Enrosadira’ were significantly brighter, and ‘Dafne’, ‘Kokanee’ and ‘Versailles’ had a more intense red color for floricanes (Table 3).

### 2.3. Fruit Weight and Textural Characteristics

Fruit weight was significantly affected by both factors and interaction at *p* ≤ 0.001 (Table 4), ranging from 3.65 g (‘Kokanee’ on floricanes) to 6.83 g (‘Dafne’ on primocanes).

‘Dafne’ and ‘Versailles’ were comparable to the control cultivar regarding fruit size, having significantly larger fruits on the primocanes similar to ‘Kokanee’ and ‘Primalba’, while ‘Enrosadira’ enlarged fruits on floricanes.

Concerning texture results (Table 4), we confirm a high dependence of texture parameters on the cultivar, as previously reported by Giongo et al. [30]. Cropping also significantly affected hardnesses 1 and 2, where fruits were firmer on floricanes in the spring crop. ‘Versailles’ differed significantly from the control cultivar for all three parameters affected by the cultivar, having the firmest fruits at both compression cycles. The fruits of ‘Dafne’ were also identified as being harder than those of the control cultivar ‘Enrosadira’, but less cohesive. In contrast, ‘Kokanee’ and ‘Paris’ were comparable to the control cultivar regarding both cohesiveness and hardness.

### 2.4. Sensory Analysis

**Figure 3:**
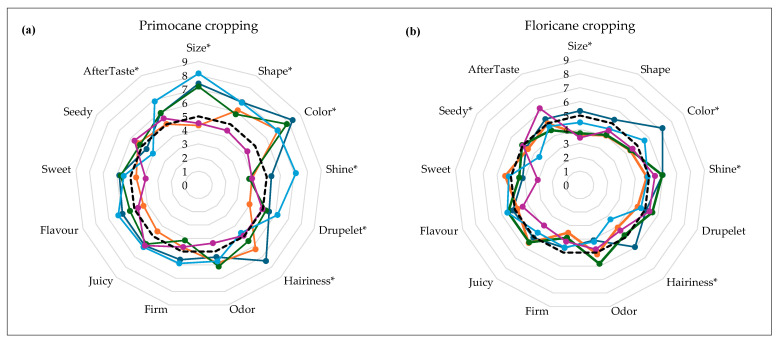
A spider web of descriptive sensory data of the five newly introduced raspberry cultivars in comparison to the control: (**a**) primocane cropping and (**b**) floricane cropping. Color code in graphs—‘Dafne’, ‘Kokanee’, ‘Paris’, ‘Versailles’, and ‘Primalba’. The control sample (‘Enrosadira’) is presented as a dotted line ▪▪▪▪. * indicates statistically significant differences among cultivars at *p* ≤ 0.05.

Sensory analysis data showed that the fruits of ‘Versailles’ and ‘Dafne’ were firmer, larger and more elongated than the control, as perceived by consumers in fall cropping (Figure 3a). Consumer acceptance is generally determined by the high intensity of raspberry aroma and flavor, color uniformity, floral aroma, green flavor and aftertaste [20]. Regarding berry color, all cultivars except ‘Primalba’ were marked as darker than ‘Enrosadira’ in the fall cropping (Figure 3a), which is opposite to the instrumentally measured values in our study (Table 3). ‘Primalba’, for most of the attributes, scored lower than the control, being smaller, flatter, slightly lighter and less sweet. Greater variations in size, shape and color between the two crops were recorded with the cultivar ‘Paris’. There were no statistically significant differences among samples for odor, firmness, juiciness, flavor and sweetness (*p* > 0.05) for both crops. This is contrary to Aaby et al.’s [29] findings, where odor and flavor showed most of the variations among raspberry samples. On average, three of the panelists identified at least one atypical taste per cultivar, not of high intensity . All samples, at least once, were identified with acid and cloying atypical taste, referring to sour and un-fresh taste, respectively. The fruits of ‘Dafne’ were additionally described as green and grassy, which was previously detected by Lippi et al. [18], but at low intensity, with acidic and fruity flavors prevailing in this cultivar. ‘Paris’ was confirmed as cloying and floral and ‘Versailles’ as cloying and acidic during both cropping seasons. Floral taste, one of the commonly associated with raspberries [18], was also recognized in cultivars ‘Kokanee’ and ‘Primalba’.

**Figure 4:**
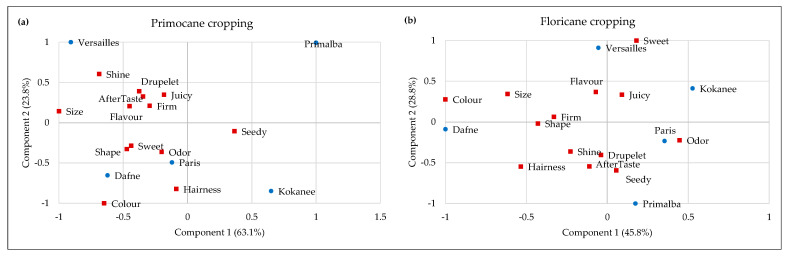
Bi-plot of descriptive sensory data of the five newly introduced raspberry cultivars: (**a**) primocane cropping and (**b**) floricane cropping.

The results of the multivariate statistical analysis, which included 13 attributes, are displayed in Figure 4. For primocane cropping (Figure 4a), analysis shows that the first two components explain 86.9% of the variance of the original dataset. As presented in the bi-plot, the main difference among different sensory attributes was observed between fruit size and seeds during mastication (component 1) and between color and hairiness on one side and shininess, drupelets, juiciness, flavor, aftertaste and firmness on the other (component 2). Regarding cultivars, ‘Paris’ and ‘Dafne’ were characterized by most of the attributes, while ‘Versailles’ was correlated with shininess and drupelets. At the same time, ‘Primalba’ and ‘Kokanee’ were ‘stand-alone’ cultivars with different sensory profiles compared to the others. The sensory profiles were slightly modified on floricane cropping (Figure 4b). ‘Dafne’ was characterized by the most pronounced color and size, ‘Versailles’ with sweetness and ‘Paris’ with odor. Similar to the primocane crop, ‘Kokanee’ and ‘Primalba’ have a slightly different sensory profile compared with the others.

### 2.5. Total Quality Index

**Figure 5:**
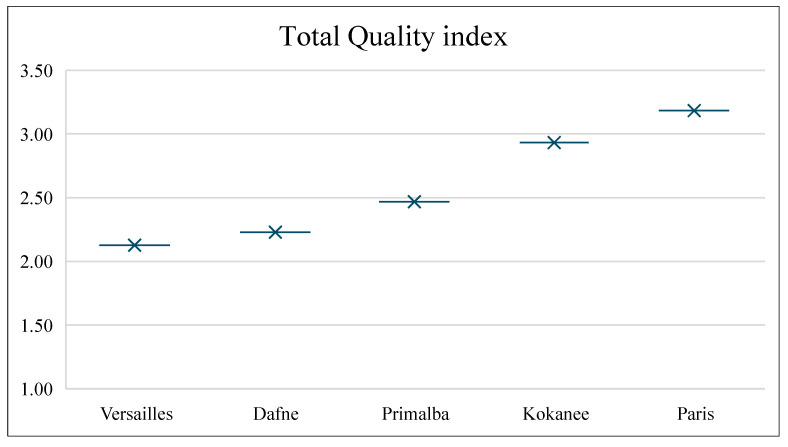
Total quality index (TQI) of different raspberry cultivars.

TQI results for all quality characteristics are presented in Figure 5. It shows that the best TQI was achieved for the cultivar ‘Versailles’, followed by ‘Dafne’, while the cultivar ‘Paris’ scored the worst. The overall fruit quality scores clearly distinguish the selected raspberry cultivars.

## 3. Materials and Methods

### 3.1. Experimental Design and Plant Material

The research was conducted in the experimental field of the company ‘Floriva’ (Ivanjica, Serbia), located in the southwestern Serbia (43°37′07.3″ N, 20°10′41.2″ E, 468 m a.s.l.) during the period 2023–2024. This area is characterized by an average annual temperature of 11.5 °C, total precipitation of 813.1 mm and relative air humidity of 81%. The average temperature (°C) and relative humidity (%) of the leaf surface of tested cultivars along with the physiological status of the plants were monitored by an MC-100 chlorophyll meter (Amtast, Lakeland, FL, USA) during both cropping seasons, and average values of the leaf surface temperature and relative humidity are presented in Table 5.

**Figure 6:**
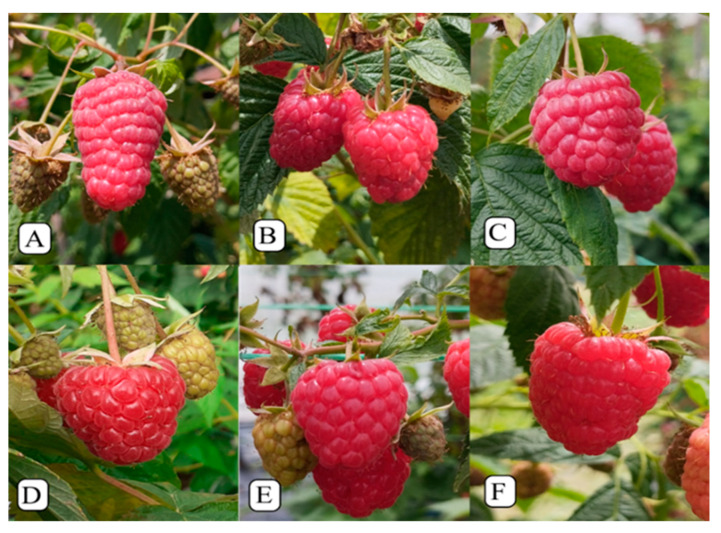
Evaluated remontant raspberry cultivars: (**A**) ‘Enrosadira’, (**B**) ‘Dafne’, (**C**) ‘Primalba’, (**D**) ‘Paris’, (**E**) ‘Versailles’ and (**F**) ‘Kokanee’.

Five newly introduced remontant cultivars ‘Dafne’, ‘Kokanee’, ‘Paris’, ‘Versailles’ and ‘Primalba’ were compared to the control cultivar ‘Enrosadira’ (Figure 6) during fall and spring cropping. Comparisons between the two croppings for each cultivar were carried out as well. The experiment was set up in 3 repetitions with 6 plants each (18 plants for each cultivar). Plug plants of the tested cultivars were received from foreign companies (Sant Orsola Agricola, Trento, Italy; Molari and Berry Lab Società Agricola, Cezena, Italy; Emcocal, Spain; Skyberry, Bulgaria; Earthmarket, France) and planted in the spring of 2022 using 10 L pots filled with coco peat (Galuku, Australia).

Pots were spaced 0.4 m apart in a row and 2.1 m between rows. Two primocanes per pot were maintained and supported with a trellis system. Plants were fertigated through drip irrigation using two emitters and a water capacity of 3 l per pot per hour, which was adjusted to the plant’s water requirements and calculated according to the evapotranspiration values. The nutrient solution consisting of NO_3_^−^ 6 mM, H_2_PO_4_^−^ 1 mM, SO_4_^2^ 1.5 mM, NH_4_^+^ 0.6 mM, K^+^ 3 mM, Ca^2+^ 4 mM, Mg^2+^ 4 mM, Fe^2+^ 30 μM, Mn^2+^ 20 μM, Zn^2+^ 8 μM, B 12 μM, Cu 1.75 mM and Mo 0.75 mM was applied following the recommendation of Valentinuzzi et al. [31]. Fertigation was carried out 4 times a week to target crops by each stage of every irrigation cycle from the end of April to the end of October in 2023 and from the end of April to the middle of July in 2024. The nutrient solution was characterized by an average Electrical Conductivity (EC) of 2000 μS cm^−1^ and a pH value of around 5.5.

A foliar spray with 1.5 m g H_3_BO_3_ plant^−1^ and amino acids, ‘Trainer’ (Hello Nature, Biandrate (NO), Italy), was used 4 times from the end of July to the end of October in 2023, and in the following season, 3 applications were carried out from the end of April to end of June.

The experimental field was covered with a white anti-hail net (mesh size of 2.8 × 8.7 mm), produced by Agrinova (Cassina de’ Pecchi (MI), Italy). Plants were maintained in a double-cropping growing system, where primocanes were retained after fall fruiting to produce fruits on floricanes in the spring of the following year. Fully ripe fruits from the fall crop covering the period from late August to the end of October 2023 and the spring crop from the beginning of June to the end of July 2024 were randomly collected in three replicates of 80 fruits each (240 fruits per cultivar). After harvest, fruit samples were immediately transported to the laboratory of the Department of Food Safety and Quality Management, Faculty of Agriculture, University of Belgrade (Serbia), where two-thirds of the fruit samples per replicates were used for evaluation of fruit weight, texture parameters, color and sensory analysis, while the rest of the fruits were frozen in liquid nitrogen and stored at −20 °C prior to chemical analysis to prevent the effect of postharvest factors.

### 3.2. Determination of Individual Sugars and Organic Acids

Before extraction, frozen fruit samples were thawed and measured. Sugars and acids were extracted by homogenizing 1 g of fruit sample with 3 mL of 80% methanol using a mortar and pestle. Extracts were centrifuged at 13,000× g for 10 min at 4 °C and filtered through 0.22 µm nylon syringe filters (Phenomenex, Torrance, CA, USA). The methanol supernatants for every cultivar were prepared in triplicate and stored at −80 °C until the analysis.

High-performance liquid chromatography (HPLC) analyses were performed on a Waters Breeze chromatographic system (Waters, Milford, MA, USA) connected to a Waters 2465 electrochemical detector with a 3 mm gold working electrode and a hydrogen reference electrode. Separation of sugars was performed on a CarboPac PA1 (Dionex, Sunnyvale, CA, USA) 250 × 4 mm column equipped with a corresponding CarboPac PA1 guard column at a constant temperature of 30 °C and at a flow rate of 1.0 mL min^−1^. Sugars were eluted isocratically using 200 mM sodium hydroxide prepared from 50% *w*/*w* low-carbonate NaOH (J.T. Baker, Deventer, The Netherland) by adding 10.5 mL to the final volume of 1 L vacuum-degassed deionized water. Signals were detected in the pulsed amperometric mode using the following waveform: E1 = +0.15 V for 300 ms; E2 = +0.75 V for 150 ms; E3 = −0.80 V for 100 ms and within 150 ms of integration time. The filter timescale was 0.5 s and the range was set to 5 µA for the full mV scale.

Separation of organic acids was performed on the same HPLC system (Waters, Milford, MA, USA) connected to a 2996 diode array detector adjusted at 210 nm. A Supelco C-610H (300 × 7.8 mm) anion exchange column and precolumn (Sigma-Aldrich, Barcelona, Spain) were used. Isocratic elution was employed with 0.1% H_3_PO_4_ as the mobile phase at a flow rate of 0.5 mL min^−1^ and at a column temperature of 40 °C. For both sugar and organic acid analysis, data acquisition and quantification by the external standard method were carried out by the Waters Empower 2 Software (Waters, Milford, MA, USA).

### 3.3. Color Measurement

The surface color was measured on a random fruit sample in three replicates using an AMT529 color spectrophotometer (Amtast, Lakeland, FL, USA and a CIE L*a*b* system (illuminant D65, observed angle 10°) in the wavelength range of 400–700 nm. L* represents lightness, ranging from black (0) to absolute white (100). Positive values at the a* axis correspond to red color and negative values to green color, while b* values represent blue for negative and yellow for positive. Chroma (C) and hue angle (h) are derived from a*b*, values, with chroma indicating color saturation and hue angle color shade. The device was calibrated with a white and black board (white board number: 284714).

### 3.4. Determination of the Fruit Weight and Texture Analysis

During the fall and spring cropping, a sample of 20 fruits per replicate was weighed on a technical scale (Acom JW-1, ACOM, Seoul, South Korea) with an accuracy of 0.01 g. Texture profile analysis (TPA) with a double compression test was performed with the Brookfield CT3 Texture Analyzer (Brookfield Engineering, Middleboro, MA, USA). The determinations of hardness 1, hardness 2, cohesiveness and springiness were conducted with the following operating parameters: trigger force 5 g/0.05 N, measurement speed 1.7 mm s^−1^, deformation depth 10 mm and 4 mm probe. Here, hardness 1 represents the peak load of the first compression cycle expressed as the maximal force (N) required to make deformation, and hardness 2 is the force (N) attained in the second compression cycle. Cohesiveness corresponds to the strength of internal connections expanded in compression. Springiness (mm) expresses the elasticity of the sample or the rate at which the deformed sample returns to its original size and shape. It also can be explained as the distance recovered by the sample between the end of the first bite and the beginning of the second [30]. Raspberry fruit was compressed in a double cycle in the equatorial position, with three replicates per cultivar.

### 3.5. Sensory Analysis Procedure

Sensory analysis was performed by a panel of five researchers from the Faculty of Agriculture with experience in evaluating different types of fruit and in descriptive analysis. All sensory tests were performed in the Laboratory for Sensory Analysis at the Faculty of Agriculture, University of Belgrade. Environmental conditions during the sensory analysis were in line with ISO 8589 [32]. Two training sessions (2 h each) were organized prior to commencing the analysis to train the panelists for main sensory attributes [33]. The sensory analysis was conducted immediately after sample collection in the fall and spring harvest. Homogeneous fruits from each cultivar were placed on plastic trays and evaluated for the attributes listed in Table S1 (Supplementary Materials), which were selected in line with the research of Lippi et al. [18]. A degree of difference test with a 9-point category-type scale, with anchors from 1 to 9, where ‘Enrosadira’, serving as a target, indicated as a ‘5’ value, was used. Five different raspberry cultivars (‘Dafne’, ‘Kokanee’, ‘Paris’, ‘Versailles’ and ‘Primalba’) were served to the assessors simultaneously in random order to enable comparing the raspberries with control samples. Sample serving procedures, instructions to panelists and palate cleansing were aligned with Tomic et al. [34]. Each panelist was provided with two samples simultaneously: one was marked as the target, and the other was coded. Random 3-digit numbers were used for the sample coding. The task was to evaluate the differences between the target sample and other raspberry cultivars. The working hypothesis was that there is no difference in overall perception between target and new cultivars. The sensory evaluation was performed in three repetitions.

### 3.6. Sweetness Index (SI)

The sweetness index was calculated according to Equation (1) as the sum of multiplied concentration values of the main individual sugars with their coefficients (glucose = 1; fructose = 2.3; sucrose = 1.35), as reported by Keutgen and Pawelzik [35].

Coefficients indicate that fructose and sucrose are 2.3 and 1.35 times sweeter than glucose, respectively.

SI = (glucose × 1) + (fructose × 2.3) + (sucrose × 1.35)(1)

### 3.7. Total Quality Index (TQI)

Quality characteristics derived during this research (individual sugars and organic acids, fruit weight, textural properties and sensory attributes) were used to calculate the total quality index (TQI), according to Equation (2) given below, in line with works of Finotti et al. [36] and Djekic et al. [37].

(2) $$TQI=\sqrt{\sum_{j=1}^{N}({{QI}_{j})}^{2}}$$

QI—quality index of each quality characteristic; N—number of quality characteristics. Rules and formulas for calculating QIs are provided in Supplementary material, Table S2.

The calculation was performed in two steps. In the first step, individual sugars and organic acids, fruit weight and textural properties were used for calculating the TQI for six cultivars. As the results confirmed that the best score was achieved for ‘Enrosadira’ , sensory attributes were added to the TQI to rank the remaining five cultivars. The rule for interpreting the TQI is ‘the lower the value, the better the TQI’ [34].

### 3.8. Statistical Analysis

Data analysis was performed using SPSS 17.0 Statistics (IBM, Armonk, NY, USA). The significant differences between the cultivars, cropping seasons and the interaction between these two factors were estimated by two-way analysis of variance at *p* ≤ 0.05, 0.01 and 0.001. The significance of the differences between the mean values was determined using the LSD test at *p* ≤ 0.05, 0.01 and 0.001. For the factor cultivar, significant differences are presented in comparison to the control cultivar ‘Enrosadira’. Data are displayed as the means ± standard errors. Descriptive sensory data were standardized among assessors and presented using a spider web chart. A one-way ANOVA was employed to analyze the differences in sensory attributes between cultivars. In addition, raw descriptive data of the sensory attributes were subjected to Principal Component Analysis (PCA).

## 4. Conclusions

The development of new cultivars that meet increased consumer preferences regarding both high nutritional and sensorial fruit qualities is the only option for the promotion of raspberry consumption. This study provides valuable insight into the fruit metabolomic, textural and sensory profiles of six perspective remontant raspberry cultivars identified on both primocanes and floricanes. Most parameters of the fruit quality were significantly affected by the cultivar, but significant variations were also found between the primocane and floricane crops within the same cultivar. Generally, fruits displayed a larger size and accumulated more sugars, being sweeter in the fall cropping on primocanes, while floricane crops exhibited more saturated, brighter red-colored and firmer fruits. The content of individual and total sugars as well as organic acids was significantly affected by the cultivar, while the sugar content significantly varied between the cropping seasons.

The results highlight the variations between newer remontant raspberry cultivars and their suitability to produce high-quality fruit in both fall and spring harvests, providing fresh, nutritious fruit for prolonged consumption in two seasons. Furthermore, the knowledge of variability in chemical, textural and sensorial fruit traits among cultivars could be exploited commercially, and these traits can be used as increasingly important criteria for identifying new high-performing cultivars for a particular environment and cultivation method. Our study revealed that cultivars ‘Enrosadira’, ‘Versailles’ and ‘Dafne’ performed best considering all parameters tested, which was confirmed by the TQI. Further research is needed to determine the plant physiology and productivity of promising cultivars and prove their field performance in a double-cropping system.

## Figures and Tables

**Table 1:** Effect of the cultivar and cropping season on the content of individual sugars and polyols in the fruit extracts of remontant raspberry cultivars in comparison to the control cultivar ‘Enrosadira’.

Individual Sugars/Polyols	Glucose (g 100 g^−1^ FW)	Fructose (g 100 g^−1^ FW)	Sucrose (g 100 g^−1^ FW)	Myoinositol (g 100 g^−1^ FW)	Arabinose (g 100 g^−1^ FW)
**Cultivar**					
Dafne	1.67 ± 0.25 ***	2.28 ± 0.32 **	2.62 ± 0.68 *	0.21 ± 0.04 ns	0.33 ± 0.08 ns
Kokanee	2.50 ± 0.10 ns	3.07 ± 0.13 ns	2.68 ± 0.23 *	0.21 ± 0.03 ns	0.30 ± 0.03 ns
Paris	2.77 ± 0.19 ns	3.42 ± 0.23 ns	2.73 ± 0.13 *	0.18 ± 0.02 **	0.29 ± 0.06 ns
Versailles	2.69 ± 0.33 ns	3.72 ± 0.49 *	1.66 ± 0.45 ns	0.28 ± 0.04 ns	0.174 ± 0.04 ns
Primalba	2.17 ± 0.22 **	2.57 ± 0.26 ns	3.03 ± 0.37 **	0.33 ± 0.05 ns	0.16 ± 0.01 *
Enrosadira	2.77 ± 0.21	3.07 ± 0.23	1.91 ± 0.26	0.27 ± 0.03	0.27 ± 0.04
***p *** **value**	<0.001	<0.001	0.001	0.001	0.007
**LSD_005_**	0.427	0.514	0.634	0.067	0.098
**Cropping**					
Primocane	2.67 ± 0.11	3.37 ± 0.17	2.69 ± 0.24	0.30 ± 0.02	0.19 ± 0.02
Floricane	2.18 ± 0.17	2.67 ± 0.19	2.19 ± 0.24	0.19 ± 0.02	0.32 ± 0.03
***p *** **value**	<0.001	<0.001	0.009	<0.001	<0.001
**Interaction**					
Dafne × P	2.20 ± 0.11 cd	2.98 ± 0.15 c	4.09 ± 0.40 a	0.29 ± 0.03 bc	0.16 ± 0.04 ef
Dafne × F	1.14 ± 0.08 e	1.58 ± 0.11 e	1.16 ± 0.09 e	0.12 ± 0.03 e	0.49 ± 0.08 a
Kokanee × P	2.48 ± 0.05 bc	3.10 ± 0.07 bc	2.23 ± 0.16 cd	0.24 ± 0.03 cd	0.26 ± 0.03 cde
Kokanee × F	2.51 ± 0.21 bc	3.05 ± 0.28 c	3.13 ± 0.22 bc	0.17 ± 0.03 de	0.34 ± 0.05 bc
Paris × P	2.47 ± 0.09 bc	3.06 ± 0.15 bc	2.61 ± 0.08 bc	0.2 ± 0.03 cde	0.19 ± 0.06 def
Paris × F	3.07 ± 0.28 ab	3.77 ± 0.34 b	2.85 ± 0.24 bc	0.16 ± 0.02 de	0.40 ± 0.07 ab
Versailles × P	3.40 ± 0.08 a	4.76 ± 0.10 a	2.60 ± 0.20 bc	0.35 ± 0.03 ab	0.08 ± 0.01 f
Versailles × F	1.98 ± 0.22 cd	2.67 ± 0.34 cd	0.72 ± 0.27 e	0.21 ± 0.03 cde	0.27 ± 0.01 bcde
Primalba × P	2.50 ± 0.30 bc	2.99 ± 0.35 c	3.23 ± 0.71 bc	0.43 ± 0.05 a	0.16 ± 0.02 ef
Primalba × F	1.84 ± 0.21 d	2.15 ± 0.20 de	2.84 ± 0.36 bc	0.24 ± 0.01 cd	0.17 ± 0.00 ef
Enrosadira × P	2.98 ± 0.29 ab	3.31 ± 0.33 bc	1.39 ± 0.27 de	0.27 ± 0.06 bc	0.32 ± 0.05 bcd
Enrosadira × F	2.57 ± 0.30 bc	2.82 ± 0.32 cd	2.43 ± 0.06 bc	0.27 ± 0.01 bc	0.22 ± 0.07 cde
***p *** **value**	0.001	<0.001	<0.001	0.043	0.002

Data are presented as the means (*n* = 3) ± standard error. The significance of the factors and their interaction effect is indicated by the *p* value. Different letters within the same column indicate statistically significant differences according to the LSD test at *p* ≤ 0.05. Differences caused by the cultivar are displayed in relation to the control cultivar ‘Enrosadira’ and indicated as follows: ns, *, ** and *** represent nonsignificant and significant differences at *p* ≤ 0.05, *p* ≤ 0.01 and *p* ≤ 0.001, respectively. FW—fresh weight; P—primocane; F—floricane.

**Table 2:** Effect of the cultivar and cropping season on the content of individual organic and total acids in the fruit extracts of remontant raspberry cultivars in comparison to the control cultivar ‘Enrosadira’.

Organic Acids	Citric (mg g^−1^ FW)	Malic (mg g^−1^ FW)	Tartaric (mg g^−1^ FW)	Shikimic (µg g^−1^ FW)	Fumaric (µg g^−1^ FW)	Total Acids (mg g^−1^ FW)
**Cultivar**						
Dafne	8.33 ± 0.30 ns	1.14 ± 0.05 ns	0.08 ± 0.01	7.05 ± 0.63 ***	6.94 ± 0.33 ns	9.56 ± 0.30 ns
Kokanee	7.20 ± 0.39 ns	1.02 ± 0.04 ns	0.06 ± 0.01	16.07 ± 2.42 ***	7.76 ± 0.87 ns	8.31 ± 0.41 ns
Paris	9.74 ± 0.24 *	1.42 ± 0.10 **	0.08 ± 0.01	6.80 ± 1.47 ***	3.58 ± 0.33 ***	11.25 ± 0.31 *
Versailles	8.15 ± 0.52 ns	1.19 ± 0.06 ns	0.08 ± 0.01	15.02 ± 2.62 ***	4.55 ± 0.39 **	9.44 ± 0.59 ns
Primalba	8.93 ± 0.63 ns	1.22 ± 0.06 ns	0.09 ± 0.01	12.25 ± 1.26 ns	7.85 ± 0.66 ns	10.26 ± 0.69 ns
Enrosadira	8.09 ± 0.42	1.17 ± 0.04	0.10 ± 0.01	11.00 ± 1.02	7.13 ± 1.23	9.38 ± 0.43
***p *** **value**	0.012	0.005	0.132	<0.001	<0.001	0.008
**LSD_0_._05_**	1.286	0.185	-	1.554	1.457	1.432
**Cropping**						
Primocane	8.4 ± 0.31	1.22 ± 0.05	0.08 ± 0.01	14.72 ± 1.22	6.12 ± 0.55	9.72 ± 0.35
Floricane	8.42 ± 0.30	1.17 ± 0.04	0.09 ± 0.01	8.00 ± 0.65	6.48 ± 0.56	9.68 ± 0.32
***p *** **value**	0.967	0.317	0.118	<0.001	0.378	0.948
**Interaction**						
Dafne × P	8.18 ± 0.33	1.16 ± 0.03	0.10 ± 0.01 ab	8.32 ± 0.50 d	7.44 ± 0.40 bc	9.45 ± 0.31
Dafne × F	8.48 ± 0.55	1.11 ± 0.10	0.07 ± 0.02 bcd	5.79 ± 0.35 e	6.43 ± 0.37 cd	9.67 ± 0.58
Kokanee × P	6.50 ± 0.25	1.04 ± 0.05	0.04 ± 0.01 d	21.30 ± 1.00 a	9.38 ± 0.77 ab	7.61 ± 0.29
Kokanee × F	7.91 ± 0.44	1.00 ± 0.07	0.08 ± 0.01 abc	10.83 ± 0.87 c	6.14 ± 0.76 cd	9.00 ± 0.51
Paris × P	9.79 ± 0.27	1.56 ± 0.17	0.05 ± 0.01 cd	10.06 ± 0.28 cd	3.27 ± 0.36 e	11.42 ± 0.43
Paris × F	9.68 ± 0.45	1.29 ± 0.07	0.10 ± 0.01 ab	3.54 ± 0.36 f	3.90 ± 0.56 e	11.08 ± 0.52
Versailles × P	8.21 ± 1.08	1.23 ± 0.13	0.06 ± 0.01 bcd	20.59 ± 1.65 a	4.49 ± 0.69 de	9.52 ± 1.22
Versailles × F	8.10 ± 0.43	1.15 ± 0.04	0.10 ± 0.02 ab	9.44 ± 0.61 cd	4.60 ± 0.52 de	9.36 ± 0.48
Primalba × P	8.90 ± 0.50	1.22 ± 0.04	0.10 ± 0.00 ab	14.90 ± 0.44 b	7.51 ± 0.52 abc	10.24 ± 0.46
Primalba × F	8.97 ± 1.33	1.21 ± 0.14	0.09 ± 0.02 abc	9.61 ± 0.86 cd	8.20 ± 1.34 abc	10.29 ± 1.48
Enrosadira × P	8.83 ± 0.45	1.10 ± 0.07	0.11 ± 0.01 a	13.17 ± 0.28 b	4.62 ± 0.51 de	10.06 ± 0.52
Enrosadira × F	7.35 ± 0.39	1.23 ± 0.02	0.09 ± 0.02 abc	8.82 ± 0.59 cd	9.64 ± 1.01 a	8.70 ± 0.43
***p *** **value**	0.385	0.400	0.031	<0.001	<0.001	0.537

Data are presented as the means (*n* = 3) ± standard error. The significance of the factors and their interaction effect is indicated by the *p* value. Different letters within the same column indicate statistically significant differences according to the LSD test at *p* ≤ 0.05. Differences caused by the cultivar are displayed in relation to the control cultivar ‘Enrosadira’ and indicated as follows: ns, *, ** and *** represent nonsignificant and significant differences at *p* ≤ 0.05, *p* ≤ 0.01 and *p* ≤ 0.001, respectively. FW—fresh weight; P—primocane; F—floricane.

**Table 3:** Interaction effect of the cultivar and cropping season on CIE Lab color parameters of raspberry fruits.

Interaction	L	a	b	C	h
Dafne × P	22.95 ± 0.63 f	14.38 ± 0.62 de	5.78 ± 0.40	15.51 ± 0.73	21.78 ± 0.46
Dafne × F	31.74 ± 0.90 c	19.03 ± 0.13 bc	7.79 ± 0.08	20.54 ± 0.17	22.17 ± 0.26
Kokanee × P	24.98 ± 0.34 de	15.4 ± 0.64 de	5.64 ± 0.57	16.43 ± 0.79	20.01 ± 1.00
Kokanee × F	35.45 ± 0.49 a	19.90 ± 0.66 ab	6.22 ± 0.71	20.88 ± 0.85	17.28 ± 1.29
Paris × P	25.55 ± 0.86 de	15.78 ± 1.67 de	6.00 ± 0.69	16.89 ± 1.78	20.93 ± 0.85
Paris × F	33.23 ± 0.60 bc	15.67 ± 0.44 de	5.17 ± 0.33	16.53 ± 0.51	18.45 ± 0.82
Versailles × P	25.05 ± 0.82 de	13.92 ± 0.70 e	3.84 ± 0.71	14.48 ± 0.82	15.09 ± 2.20
Versailles × F	35.01 ± 0.84 ab	17.45 ± 1.64 bc	5.77 ± 0.88	18.32 ± 1.86	18.12 ± 0.92
Primalba × P	25.54 ± 0.70 de	19.52 ± 0.41 ab	8.39 ± 0.51	21.26 ± 0.57	23.13 ± 0.93
Primalba × F	26.41 ± 0.10 d	22.22 ± 0.60 a	9.21 ± 0.69	24.08 ± 0.80	22.32 ± 1.14
Enrosadira × P	23.62 ± 0.54 ef	16.72 ± 1.45 cd	6.19 ± 0.70	17.83 ± 1.60	20.11 ± 0.68
Enrosadira × F	25.27 ± 0.71 de	16.11 ± 0.77 de	6.48 ± 0.21	17.37 ± 0.78	21.95 ± 0.28
***p *** **value**	<0.001	0.032	0.186	0.054	0.056

Data are presented as the means (*n* = 3) ± standard error. Different letters within the same column indicate statistically significant differences according to the LSD test at *p* ≤ 0.05. The significance of the interaction effect is indicated by the *p* value. P—primocane; F—floricane.

**Table 4:** Effect of the cultivar and cropping season on fruit weight and textural traits of remontant raspberry cultivars in comparison to the control cultivar ‘Enrosadira’.

	Fruit Weight (g)	Hardness 1 (N)	Hardness 2 (N)	Cohesiveness	Springiness (mm)
**Cultivar**					
Dafne	6.11 ± 0.34 ns	0.87 ± 0.10 **	0.30 ± 0.06 ns	0.15 ± 0.02 **	4.61 ± 0.29
Kokanee	4.14 ± 0.27 ***	0.74 ± 0.04 ns	0.35 ± 0.04 ns	0.17 ± 0.01 ns	4.66 ± 0.24
Paris	5.00 ± 0.07 ***	0.69 ± 0.07 ns	0.36 ± 0.05 ns	0.22 ± 0.03 ns	5.15 ± 0.78
Versailles	5.59 ± 0.41 ns	1.08 ± 0.11 ***	0.42 ± 0.07 *	0.15 ± 0.01 **	5.01 ± 0.27
Primalba	5.31 ± 0.23 **	0.64 ± 0.10 ns	0.20 ± 0.03 ns	0.14 ± 0.01 **	3.76 ± 0.20
Enrosadira	5.97 ± 0.22	0.59 ± 0.08	0.30 ± 0.03	0.21 ± 0.02	4.18 ± 0.19
***p *** **value**	<0.001	<0.001	0.008	0.002	0.089
**LSD_0_._05_**	0.418	0.190	0.107	0.046	-
**Cropping**					
Primocane	5.71 ± 0.19	0.65 ± 0.05	0.28 ± 0.02	0.18 ± 0.02	4.77 ± 0.3
Floricane	5.00 ± 0.21	0.88 ± 0.05	0.36 ± 0.03	0.16 ± 0.01	4.35 ± 0.13
***p *** **value**	<0.001	<0.001	0.009	0.068	0.153
**Interaction**					
Dafne × P	6.83 ± 0.19 a	0.65 ± 0.07	0.18 ± 0.03 d	0.13 ± 0.02 de	4.20 ± 0.34
Dafne × F	5.38 ± 0.09 bcd	1.08 ± 0.05	0.41 ± 0.05 abc	0.16 ± 0.03 cde	5.02 ± 0.37
Kokanee × P	4.63 ± 0.34 e	0.76 ± 0.09	0.40 ± 0.06 abc	0.18 ± 0.02 bcd	4.97 ± 0.25
Kokanee × F	3.65 ± 0.04 f	0.72 ± 0.01	0.31 ± 0.02 cd	0.16 ± 0.01 cde	4.35 ± 0.34
Paris × P	5.13 ± 0.07 cde	0.60 ± 0.10	0.27 ± 0.04 cd	0.24 ± 0.06 ab	6.09 ± 1.46
Paris × F	4.86 ± 0.05 de	0.79 ± 0.06	0.46 ± 0.02 ab	0.21 ± 0.03 abc	4.20 ± 0.10
Versailles × P	6.37 ± 0.28 a	1.01 ± 0.12	0.32 ± 0.05 bcd	0.14 ± 0.003 de	5.45 ± 0.30
Versailles × F	4.82 ± 0.4 de	1.15 ± 0.19	0.52 ± 0.11 a	0.16 ± 0.01 cde	4.57 ± 0.28
Primalba × P	5.77 ± 0.18 b	0.42 ± 0.04	0.19 ± 0.02 d	0.17 ± 0.01 ab	3.45 ± 0.10
Primalba × F	4.86 ± 0.17 de	0.86 ± 0.04	0.21 ± 0.05 d	0.10 ± 0.00 abc	4.06 ± 0.33
Enrosadira × P	5.5 ± 0.15 bc	0.48 ± 0.03	0.33 ± 0.04 bcd	0.26 ± 0.01 a	4.46 ± 0.19
Enrosadira × F	6.45 ± 0.06 a	0.70 ± 0.13	0.27 ± 0.05 cd	0.16 ± 0.01 cde	3.89 ± 0.24
***p *** **value**	<0.001	0.124	0.012	0.047	0.108

Data are presented as the means (*n* = 3) ± standard error. The significance of the factors and their interaction effect is indicated by the *p* value. Different letters within the same column indicate statistically significant differences according to the LSD test at *p* ≤ 0.05. Differences caused by the cultivar are displayed in relation to the control cultivar ‘Enrosadira’ and indicated as follows: ns, *, ** and *** represent nonsignificant effects and significant effects at *p* ≤ 0.05, *p* ≤ 0.01 and *p* ≤ 0.001, respectively. P—primocane; F—floricane.

**Table 5:** The average temperature and relative humidity of the leaf surface of tested raspberry cultivars during primocane (2023) and floricane cropping (2024) seasons.

Leaf Parameters/Cropping	Primocane Cropping		Floricane Cropping	
	August	September	June	July
Temperature (°C)	31.72	30.89	28.20	33.35
Relative Humidity (%)	63.52	66.93	63.88	60.64

## Data Availability

The data presented in this study are available in the article and the Supplementary Materials.

## Supplementary Materials

The following supporting information can be downloaded at https://www.mdpi.com/article/10.3390/plants13233382/s1: Table S1: Scale degree and direction of differences in comparison to the control sample (‘Enrosadira’) and description of evaluated sensory attributes; Table S2: Rules applied for calculating quality indices.
