# Supplementary material for: Variation in Chemical, Textural and Sensorial Traits Among Remontant Red Raspberry (Rubus idaeus L.) Cultivars Maintained in a Double-Cropping System
Source: Plants (Basel). 2024 Dec 1;13(23):3382. doi: 10.3390/plants13233382 (PMC11644389; doi:10.3390/plants13233382)
Supplement: Supplementary file 1 [file plants-13-03382-s001.zip › plants-3275799-supplementary.pdf]

Supplementary Materials

Variation in Chemical, Textural and Sensorial Traits Among Remontant Red Raspberry (*Rubus idaeus* L.) Cultivars Maintained in a Double-Cropping System

Slavica Spasojević [1], Vuk Maksimović [2], Dragica Milosavljević[2], Ilija Djekić[3], Dragan Radivojević[1], Ana Sredojević[3] and Jasminka Milivojević[1]

[1] Department of Fruit Science, Faculty of Agriculture, University of Belgrade, 11080 Belgrade, Serbia; slavica.spasojevic@agrif.bg.ac.rs (S.S.); draganr@agrif.bg.ac.rs (D.R.); jasminka@agrif.bg.ac.rs (J.M.)
[2] Department of Life Sciences, Institute for Multidisciplinary Research, University of Belgrade, 11030 Belgrade, Serbia; maxivuk@imsi.rs (V.M.); dragicar@imsi.rs (D.M.)
[3] Department of Food Safety and Quality Management, Faculty of Agriculture, University of Belgrade, 11080 Belgrade, Serbia; idjekic@agrif.bg.ac.rs (I.D.); ana.sredojevic@agrif.bg.ac.rs (A.S.)
* Correspondence: jasminka@agrif.bg.ac.rs

Table S1. Scale degree and direction of differences in comparison to the control sample ('Enrosadira') and description of evaluated sensory attributes.

Unacceptable difference	Significant difference	Marginal difference	Small difference	Control sample	Small difference	Marginal difference	Significant difference	Unacceptable difference
<<<less than<<<					>>>more than>>>			
1	2	3	4	5	6	7	8	9

**Attributes**	**Intensity**	**Description**
**Appearance**		
Size	small/large	Size of the berry fruit
Shape	flatter/elongated	Berry dimension evaluation
Color	brighter/darker	Intensity of red color
Shine	matte/shiny	Surface shining intensity
Drupelet uniformity	less/more	Uniformity of drupelets
Surface hairiness	less/more	Hair presence on the surface
**Texture/mouthfeel**		
Firm	soft/firm	Compression force between tongue and palate
Seedy	less/more	Sensation of seed presence
Juicy	dry/juicy	The presence of juice in the sample
**Flavor**		
Raspberry flavor	weaker/stronger	Flavor of fresh raspberry
Raspberry odor	less/more	Fruit fragrance intensity
**Taste**		
Sweet	less/more	Sweetness intensity
Aftertaste	less/more	Intensity of taste after consumption
Atypical taste	defects-9	Intensity of atypical taste
Atypical taste catalog	1-acid; 2-green; 3-chemical; 4-cloying; 5-grassy; 6-floral; 7-unripe/overripe; 8-tangy; 9-bitter; 10-fermented; 11-watery;	

<u>Description of atypical taste attributes:</u>

Acid – sharp flavor, sour taste

Green – flavor of freshly cut green grass

Chemical – flavor of chemicals

Cloying – un-fresh, sickening flavor

Grassy – green, grassy and leafy-like aroma

Floral – associated with flower flavor

Unripe/overripe – immature/too mature, starting to decay

Tangy – strong and sharp flavor

Bitter - astringent

Fermented – flavor associated with fermented, rotted fruit

Watery – watery taste, tasteless

Table S2. Rules applied for calculating quality indices.

Quality characteristic	Rule of the thumb	Formula
Organic acids (citric, malic, tartaric, shikimic, fumaric, total); cohesiveness	The nearer to the target value, the better the quality	$QI = \left\|\frac{2 * (x_i - T)}{x_{max} - x_{min}}\right\|$
Individual sugars (glucose, fructose, sucrose, myoinositol, arabinose), fruit weight, hardness, springiness, sensory product quality	The higher the value, the better the quality	$QI = \frac{\|x_{max} - x_i\|}{x_{max} - x_{min}}$

Legend:

QI – quality index; x_i – measured value of the quality characteristic; T - target value (average value in the subset of values); x_{max} – maximal value of the quality characteristic; x_{min} – minimal value of the quality characteristic.

Sensory product quality - The overall Sensory product quality is the average score of all 13 sensory attributes (Appearance - Size, Shape, Color, Shine, Drupelet uniformity, Surface hairiness, Texture/mouthfeel - Firm, Seedy, Juicy, Flavor - Raspberry flavor, Raspberry odor, Taste - Sweet, Aftertaste) using the following equation:

$$Sensory\ product\ quality = \frac{\sum_{i=1}^{M} \frac{5 - |(5 - S_i)|}{5}}{M}$$

S_i – score achieved during sensory panel using scale degree and direction of differences (Table S1) for the i-th sensory attribute; M – number of sensory attributes
